# An evaluation method for safety applied to public sports facilities in urban communities

**DOI:** 10.1016/j.mex.2025.103256

**Published:** 2025-03-08

**Authors:** Xingyue Wu, Yuan Lu, Chuyuan Ma

**Affiliations:** a School of Safety Science and Emergency Management, Wuhan University of Technology, Wuhan 430070, PR China; b China University of Petroleum (Beijing) at Karamay, Karamay 834000, PR China

**Keywords:** Urban community, Public sports facilities, Index system construction, Safety evaluation, Safety evaluation technology based on ahp and Delphi method

## Abstract

Public sports facilities' safety evaluation index system in urban communities is not perfect, and accidents occur frequently in community physical exercise. To reduce the safety risk of public sports facilities in urban communities, this study uses the analytic hierarchy process (AHP) and delphi method to examine the risk factors that have an impact on the security of public sports facilities in urban communities. We build a safety evaluation index system of public sports facilities in urban communities, which contains 4 first-level indicators, 9 s-level indicators, and 49 third-level indicators, based on the accident system elements of the safety system theory, namely ‘equipment-man-management-environment’. Empirical research was conducted by randomly selecting two communities as case studies. The results show that: Among the first-level indicators, the highest weight coefficients are equipment factor (0.4724) and personnel factor (0.2527), followed by management factor (0.1761) and environmental factor (0.0988). This evaluation system is an effective tool for evaluating public sports facilities' safety in communities.

• The present situation of public sports facilities in urban communities is investigated on the spot, collect and analyze accident cases, the safety problems of community public sports facilities are summarized.
• A safety evaluation model is developed successfully. Security and sports experts are combined to study the index system to ensure the scientificity and effectiveness of the index
• The public sports facilities in Community A, located in the main urban district, and Community B, situated in a suburban area, are selected as case study subjects for empirical application. A safety evaluation is conducted on the public sports facilities in these two communities, resulting in safety rating classifications for each.

## Graphical abstract

**Figure:**
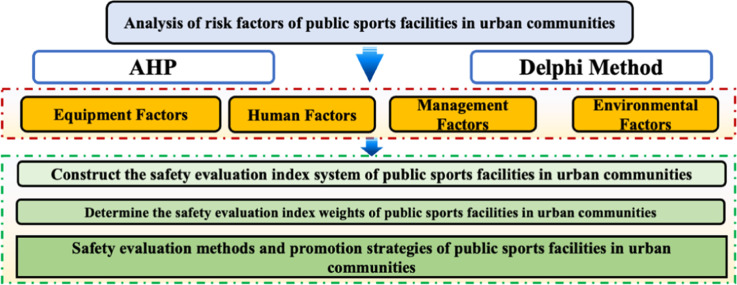

Specifications table

**Table:** 

Subject area:	Mathematics and Statistics
More specific subject area:	Safety science and engineering
Name of your method:	Safety evaluation technology based on ahp and Delphi method
Name and reference of original method:	None
Resource availability:	Software:Spss 27.0 The data are available with this article.

## Background

Community sports facilities, vital for residents' physical activities, primarily offer open spaces for daily fitness needs. They exhibit clear public and welfare characteristics, symbolizing the modern city's civilization level [1]. During the ``14th Five-Year'' period in china, urban community sports have shown remarkable development. According to the ``2022 National Sports Venues Statistical Survey Data'' released by the State General Administration of Sport, there are 4226,800 national sports venues with a total stadium area of 3702 million square meters and a per capita sports field area of 2.62 square meters [2]. Additionally, the ``2020 National Fitness Activity Survey Communique'' reports that 37.2 % of the population regularly engages in physical exercise [3]. Public sports facilities now serve as the cornerstone of healthy life in our country, and community public sports facilities are pivotal government projects in the ``sports power'' construction [4]. Public sports facilities, while enhancing people's spiritual and cultural well-being, present a direct risk to lives and property safety. The issue of ``old, damaged, missing'' facilities in urban communities is particularly severe in some areas, with equipment like horizontal bars and parallel bars degraded to the point of serving as ``clothes hangers'' [5]. Moreover, frequent injury incidents occur in community sports exercises, causing serious harm to families and individuals. The majority of these accidents result from issues related to facility layout planning, facility infrastructure, management and maintenance, and unauthorized facility usage. Regardless of the specific cause, the safety concerns arising from these incidents warrant our attention.

In terms of safety in community sports facility research, Radelet et al. [6] and Emery et al. [7] explore the causes of sports injuries resulting from community fitness activities, advocating for effective exercise interventions to prevent injuries. Finch [8] employing the health behavior intervention theory, proposes the RE-AIM intervention model tailored to residents to reduce risks. Wei [9] recommends government leadership to increase equipment usage and residents' safety awareness. Wang [[10], [11]] analyzes China's public sports facility safety management and service status from the perspectives of safety (individual sports safety, public sports venue safety, community sports facility safety) and services (service facility planning, service performance).

Presently, research on the safety of public sports facilities in urban communities predominantly focuses on the macroscopic level. This study focuses on constructing a safety evaluation system for public sports facilities in urban communities. The objective is to develop an operational evaluation model and establish the safety evaluation index system through quantitative and qualitative analyses, employing the safety systems engineering method. The study quantifies and evaluates the probability of injury accidents during community sports activities. Based on the evaluation results, management departments can be guided to comprehend safety risks, formulate management measures, clarify responsibilities, and ensure safe, healthy, and harmonious urban community sports activities.

## Method details

### Delphi method

To quantitatively evaluate the safety of public sports facilities in urban communities in China, a preliminary evaluation index was developed through literature review, expert interviews, and field research. The initial indicator system underwent evaluation and design using the five-component LIKERT method to assess the significance of each index. Subsequently, a first round of expert consultation questionnaires was created and distributed to relevant experts and government departments. After statistical data correction, second and third rounds of questionnaires were generated. Following three rounds of revision, expert opinions converged, leading to the establishment of the safety evaluation index for public sports facilities in urban communities. The Delphi method uses the following steps to establish evaluation indicators [12,13]. Delphi experts are listed in Table 1.

**Table 1:** Delphi survey expert.

Number of experts	Discipline or field of work	Title/position	Average years of relevant research
5	Sports management	4 professors, 1 associate professor	21.8
3	Sports government department	Division level leader 1 person, director 2 person	28
8	Safety engineering	6 professors, 2 associate professors	20
2	Material science	1 professor, 1 associate professor	10
1	Sport equipment	manager	10
1	Community manager	director	16

Reliability test: SPSS 27.0 software was employed to assess the reliability of the consultation questionnaire in this study. The Cronbach coefficient method was used to examine questionnaire consistency. A Cronbach's α > 0.6 indicates high reliability, confirming the reasonableness of the questionnaire design. The results indicated Cronbach's α values of 0.865, 0.943, and 0.978 for the primary, secondary, and tertiary indexes in the internal consistency test, respectively. Note: The questionnaire's reliability satisfies the criteria.

Validity Test: The questionnaire's validity was assessed using the factor analysis method to obtain the KMO value and Bartlett sphericity test. The results revealed a KMO value of 0.730 and a significant Bartlett sphericity test (*P* < 0.01), indicating good questionnaire validity.

Expert positive coefficient, authority coefficient and coordination coefficient

(1) The expert positive coefficient is commonly indicated by the investigation's recovery rate. A higher recovery rate signifies greater interest and active participation from experts in the research. In this study, a total of 20 questionnaires were distributed across three rounds, and all 20 were returned, resulting in a positive coefficient of 100 %, highlighting the experts' strong support for the study.
(2) The authority of experts is mainly determined by the expert judgment coefficient and the degree of familiarity with the consultation content. The calculation formula is as follows:
  C_r_ = (C_s_+C_a_)/2

Ca represents the foundation for item judgment, as illustrated in Table 2. An expert authority coefficient Cr≥0.7 indicates reliable consulting results, with a higher coefficient signifying a greater degree of authority. In the initial questionnaire round, the study developed the expert judgment basis questionnaire and included options to assess familiarity with the survey content. The corresponding quantitative values of the two are as follows:

**Table 2:** Criteria and quantification of expert authority coefficient calculation and familiarity level measurement in this study.

Reasons of judgment	quantity value			degree of familiarity	quantity value
practical experiences	0.5	0.4	0.3	very familiar	0.9
theoretical analysis	0.3	0.2	0.1	quite familiar	0.7
Refer to domestic and foreign literature	0.1	0.1	0.1	General familiarity	0.5
intuitive feeling	0.1	0.1	0.1	unfamiliar	0.3
				Very unfamiliar	0.1

Based on the questionnaire survey, the expert judgment coefficient was 0.925, the familiarity with the consultation content was 0.732, and the expert authority coefficient was 0.83. Following the authority coefficient criteria mentioned above, the experts consulted in this study are deemed to possess good authority.

**Fig. 1:**
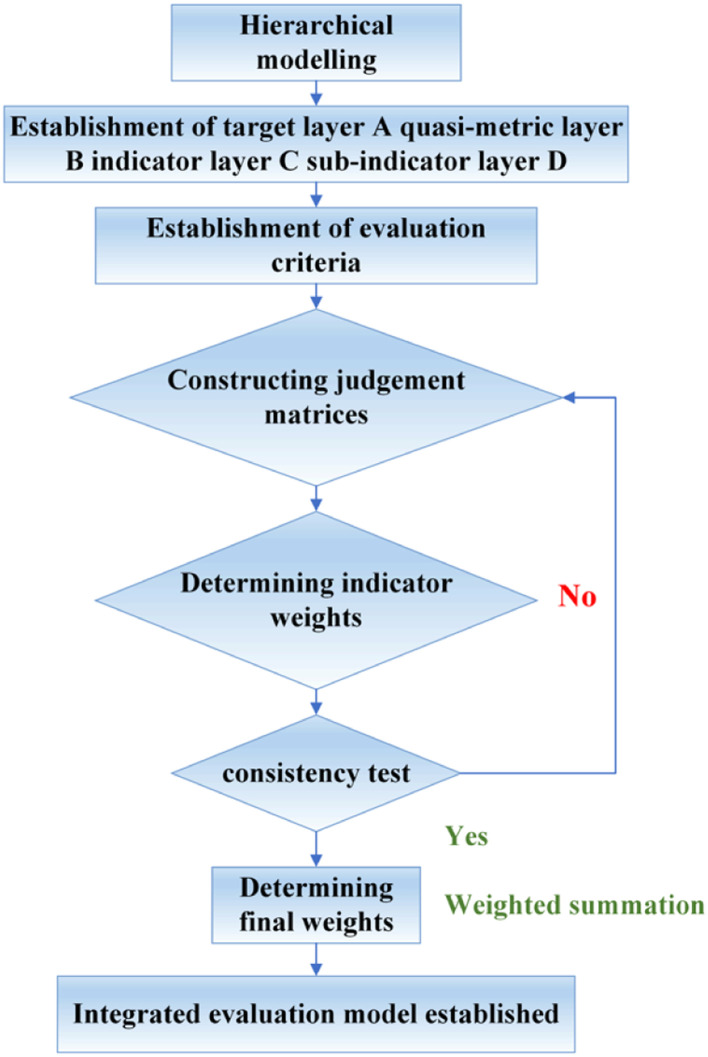
Steps for calculating weights.

**Fig. 2:**
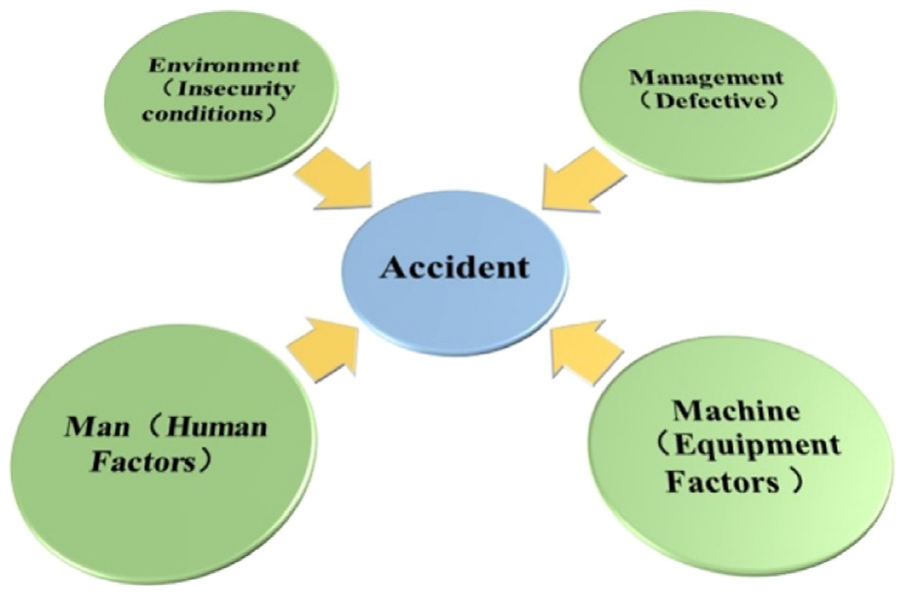
Schematic diagram of ``4M'' elements of the accident system.

AHP is a decision analysis method that combines qualitative and quantitative analysis proposed by Saaty [14]. Experts are invited to compare the importance of the evaluation indicators between each level of the constructed evaluation index system of public sports facilities in urban communities two by two, firstly, to construct a comparative judgment matrix, and secondly, to determine the weight of the indicators using mathematical methods [[15], [16], [17]] to determine the weight of the evaluation indicators, and the flow chart of the hierarchical analysis method, shown in Fig. 1, and the investigating experts are the 15 experts selected in Table 1. Fig. 2

### Establishment of safety evaluation index system

As described above, based on the systematic and comprehensive safety assessment of urban community public sports facilities, adhering to the principles of scientific rigor, systematicity, specificity, comprehensiveness, and operability, this study adopts the fundamental framework of the accident system's four elements: ``equipment—people—management—environment.'' Integrating insights from relevant literature [[18], [19], [20], [21]], along with field investigations and industry standards and norms, the ``Safety Assessment Indicator System for Urban Community Public Sports Facilities'' is established as the ultimate goal layer A. The relationships among various factors for evaluation are categorized into four hierarchical levels, preliminarily constructing an assessment system comprising four criteria layers—"facility factors, personnel factors, management factors, environmental factors"—and a total of 9 element layers and 51 indicator layers. The screening steps of the safety evaluation index system are as follows:

(1) Initial round of index selection. The expert questionnaire was distributed, and 20 experts were invited to assess the evaluation indicators. SPSS 27.0 software was utilized to statistically analyze the questionnaire results from experts. Index values with an average score of ≥3.50 and a coefficient of variation <0.25 were retained. Indicators such as ``presence of professional security personnel,'' ``implementation of the performance evaluation system,'' and ``establishment of the health management system'' did not meet the retention criteria and were removed. The indicator ``is the layout of facilities reasonable'' was modified to ``is the layout planning of facilities reasonable''; under the user category, the query regarding whether the user has a sense of security was adjusted to whether the user has a sense of self-protection. Additionally, the indicator ``Good health'' was added to the ``Internal Environment'' category. Following the initial expert feedback, adjustments were made to the index system, and the second round of expert consultation questionnaires was obtained and distributed.
(2) The second phase of index refinement. After recovering the questionnaires, data analysis was performed. For the index ``Whether community managers are insured,'' the average value, standard deviation, and coefficient of variation were 3.5, 1.069, and 0.305, respectively, leading to its removal. Within the ``facility structure'' category, two three-level indicators were introduced: ``whether the equipment meets the stability requirements'' and ``whether the static compliance of the main components meets the standards.'' In the ``user'' category, three indicators were included: ``Whether the dress is suitable for sports'' and ``whether the warm-up preparation before sports.'' Following the insights from the second round of expert feedback, adjustments were made to the index system, and the third round of expert consultation questionnaires was obtained and distributed.
(3) The third phase of index refinement. Following the recovery and processing of data from the third round of expert questionnaires, no suggestions for modifying individual indices were provided by the experts. The experts demonstrated high consistency and a relatively strong degree of coordination. Ultimately, the components of the safety evaluation index system for public sports facilities in urban communities were established, as presented in Table 5 below.

**Fig. 3:**
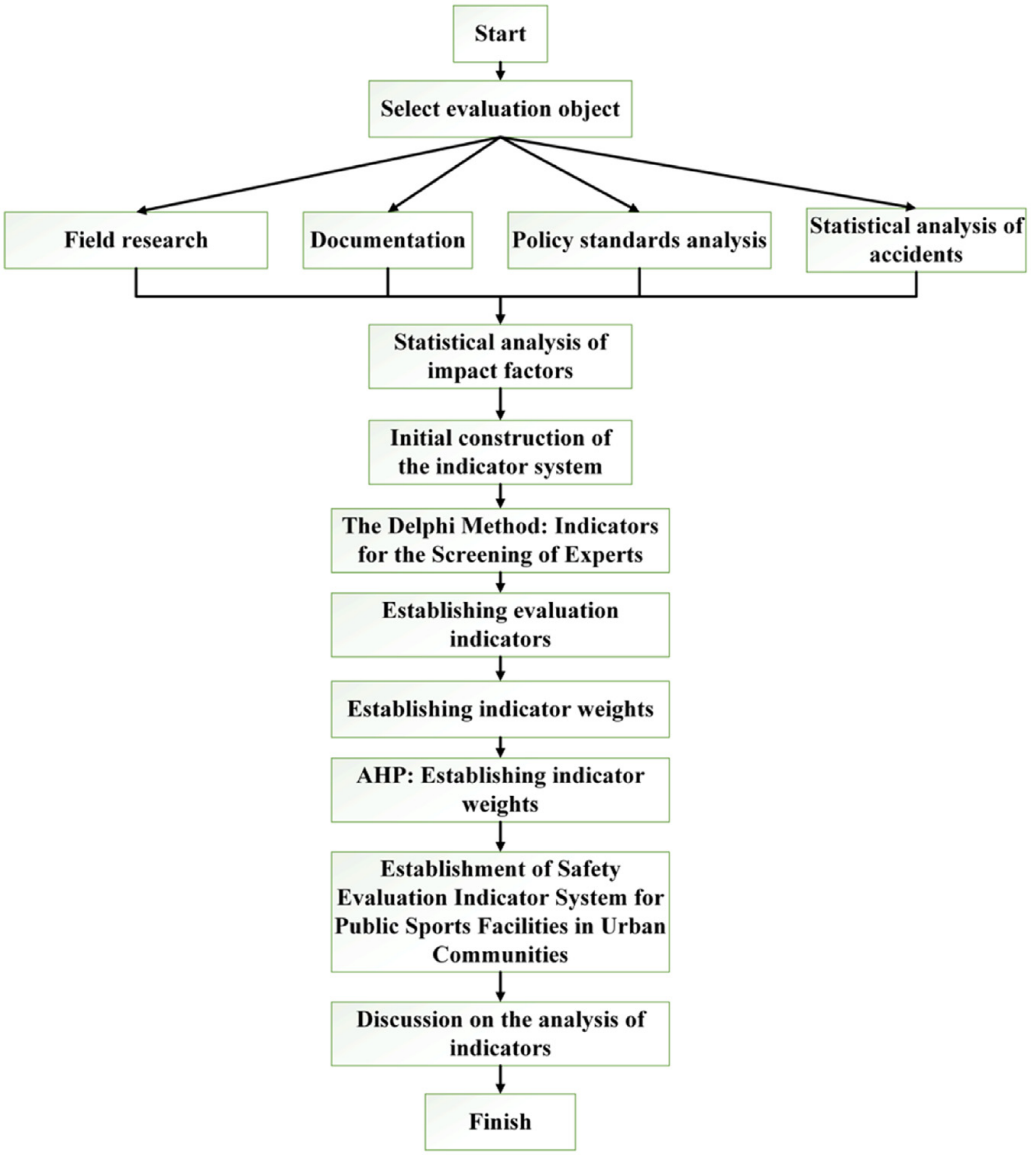
Process of establishing a safety evaluation system for public sports facilities in urban communities.

A panel of 15 experts from Table 1 was selected, and the questionnaire for evaluating the weights of safety assessment indicators for urban community public sports facilities was distributed to these experts. The AHP questionnaire employed a ``1–9 scale'' to establish a relationship matrix, prompting experts to assign values to each indicator. After the collection of expert responses, the following steps, as outlined in previous studies [[22], [23]], were utilized to calculate the weighted indices from the 15 experts. Subsequently, the average values were computed to obtain comprehensive weight coefficients. This methodology, while examining judgment matrices, allows for better quantification of the fuzzy judgments between the two factors. To illustrate, the calculation of indicator weights is demonstrated using the scores provided by a specific expert. The process of establishing a safety evaluation system for public sports facilities in urban communities is shown in Fig. 3.

**Fig. 4:**
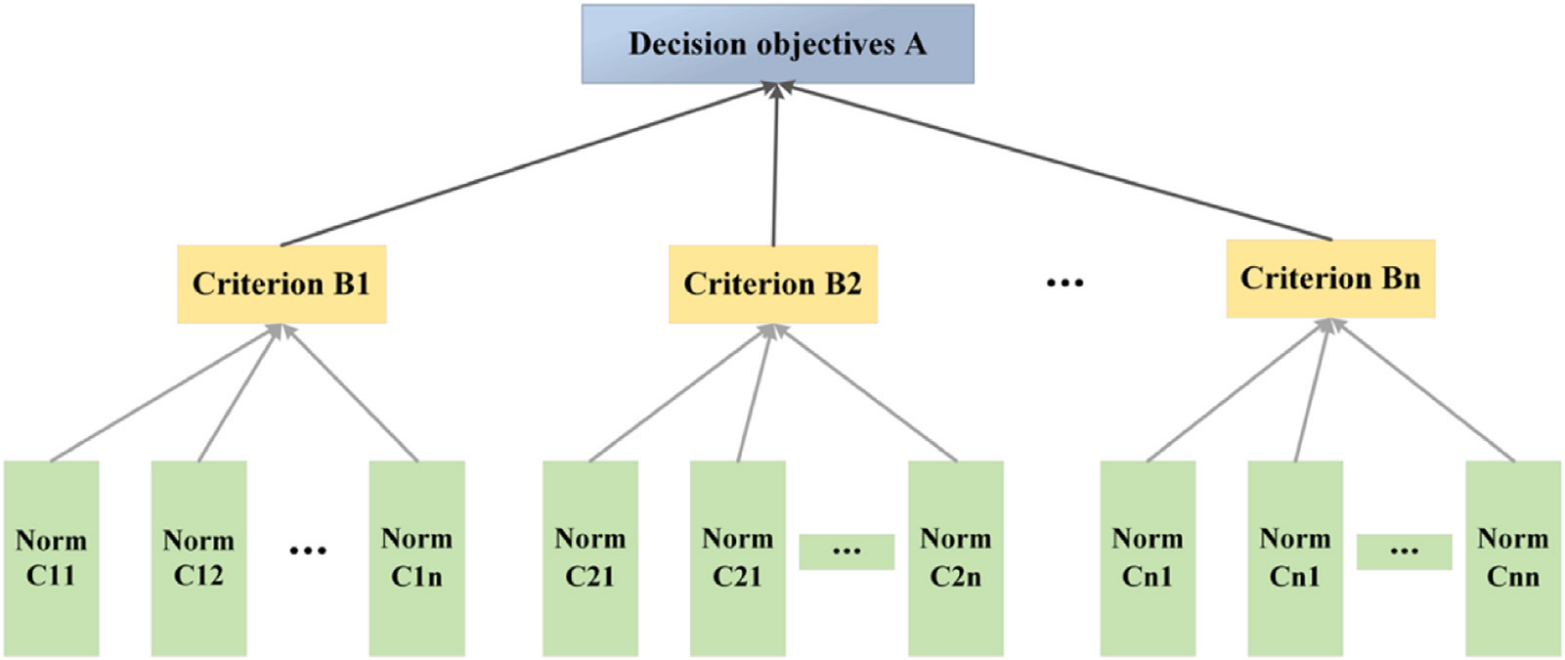
Schematic diagram of hierarchy.

A multi-level structural model of the index system was built, as shown in Fig. 4. According to the relationship between evaluation objectives, factors, and objects, they should be divided into the highest, middle, and lowest levels.

On the basis of the established index system for the safety evaluation of public sports facilities in urban communities, a pairwise comparison of the first-level indicators is carried out to construct a judgment matrix B. The calculation is performed using the widely adopted nine-point scale illustrated in Table 3.

$$B=\left(\begin{array}{cc} \begin{array}{cc} 1 & 3\\ 1/3 & 1 \end{array} & \begin{array}{cc} 5 & 7\\ 3 & 5 \end{array}\\ \begin{array}{cc} 1/5 & 1/3\\ 1/7 & 1/5 \end{array} & \begin{array}{cc} 1 & 3\\ 1/3 & 1 \end{array} \end{array}\right)$$

**Table 3:** 1–9 Meaning of scale.

Judgment scale	meaning
1	The two elements are of equal importance
3	Compared with the two elements, the former is slightly more important than the latter
5	Compared with the two elements, the former is obviously more important than the latter
7	Comparing the two elements, the former is more important than the latter
9	Comparing the two elements, the former is more important than the latter
2, 4, 6, 8	The median value of the above adjacency judgments
1/n (reciprocal)	Compared with the two elements, the latter is more important than the former described above

Calculate the eigenvector and the weight value:

(1) Initially, calculate the product of the values in each row of the matrix:
  (1) $$\text{Mi}=\prod_{j=1}^{n}\text{aij}\left(j=1,2,3\cdots n\right)$$
  (2) M1=1 × 3 × 5 × 7 = 105
(2) I am raising the product to the power of N:
  (3) $$\bar{Wi}=\sqrt[{n}]{Mi}$$
  (4) $$\bar{W1}=\sqrt[{4}]{105}=3.20109$$
(3) Normalization gives the eigenvector W=[W1,W2,…Wn], the formula is as follows:
  (5) $$\text{Wi}=\frac{\bar{\text{Wi}}}{\sum_{j=1}^{n}\bar{\text{wi}}}$$
  (6) Wi=0.5650
(4) Calculate the maximum eigenroot value (λmax)of the judgment matrix. Firstly, the λ value of each index is calculated, and then the λ_max_ value of the judgment matrix is calculated as follows:
  (7) $$\lambda i=\sum_{i=1}^{n}\frac{aij*wj}{wi}$$
  (8) $$\lambda max=\sum_{i=1}^{n}\frac{\lambda i}{n},n=Matrix\;index\;total$$

Calculate according to the formula:

(9) $$\begin{array}{c} \lambda 1=\left(1\;\times \;0.5650+3\;\times \;0.2622+5\;\times \;0.1175+7\;\times \;0.0553\right)/0.5650=4.1170\\ \lambda max=4.1170 \end{array}$$

(5) Consistency check. First, the consistency index CI is calculated.
  (10) $$CI=\frac{\lambda max-n}{n-1}=\left(4.1170-4\right)/3=0.039$$

According to the RI value table of the average consistency index, when *n* = 4, RI=0.9, enter the formula:

(11) $$CR=\frac{CI}{RI}=\frac{0.039}{0.9}=0.0438$$

Based on the calculated results, CR=0.0438<0.1, indicating that the consistency of the judgment matrix is satisfactory, allowing its use in decision analysis. Subsequently, following the same calculation procedure, weights for secondary and tertiary indicators are successively determined, and the weight coefficients for each indicator are presented in Table 4.

**Table 4:** Urban community public sports facility safety assessment index system.

destination layerA	Criterion layer B (Weight)	Element layer C (Weight)	Index layer D	weight
Safety evaluation index system of urban community public sports facilities	B1equipment factor (0.4724)	C1infrastructure (0.1868)	D1 Whether the ground condition of the site is safe, such as smooth, non-slip, etc	0.0622
			D2 Whether the facility layout planning is reasonable	0.0357
			D3 Whether the site safety buffer zone meets the general requirements of the project rules	0.0407
			D4 Whether the selection of surface layer materials meets the standard	0.0303
			D5 Whether the per capita physical exercise area and equipment meet the standard	0.0169
		C2 Facility structure (0.1982)	D6 Whether the overall structure of the instrument is intact	0.0459
			D7 Whether the surface of the instrument is safe, such as fading, edges, sharp corners, etc	0.0152
			D8 Whether the key components of the equipment are in good condition	0.0342
			D9 Whether the vulnerable parts of the equipment are in good condition	0.0179
			D10 Ageing of facilities	0.0252
			D11 Whether the gap of human body easy to contact area is qualified	0.0171
			D12Whether the NSCC authentication identifier exists	0.0133
			D13 Whether the device is configured with information, such as usage instructions, service life, etc	0.0088
			D14 Whether the equipment meets the stability requirements	0.0271
			D15 Whether the static load of the main parts meets the standard	0.0161
		C3 Security facilities (0.0740)	D16 Whether the security facilities are set up properly	0.0273
			D17 Whether the structure of security facilities is reliable	0.0200
			D18 Safety signs and signage are set correctly	0.0194
			D19 Whether to set a safe area for users to rest and change clothes	0.0109
	B2 human factor (0.2527)	C4 staff (0.0697)	D20 Have relevant professional knowledge and practical ability	0.0265
			D21 Have a high level of safety awareness	0.0170
			D22 Whether to attend safety training regularly	0.0127
			D23 Have emergency handling ability	0.0160
		C5 user of service (0.1748)	D24 The state of health is good	0.0502
			D25 Whether you have a sense of self-protection	0.0164
			D26 Compliance with rules and safety guidelines	0.0296
			D27 Whether the exercise load is reasonable	0.0239
			D28 Whether they have the ability to respond to emergencies	0.0240
			D29 Whether there is a sense of supervision and mutual assistance	0.0164
			D30 Whether you dress appropriately for sports	0.0102
			D31 Whether to warm up before exercise	0.0121
	B3 manage factor (0.1761)	C6 institutional system (0.0990)	D32 Whether there is a safety inspection system	0.0176
			D33 Whether there is a equipment management system	0.0194
			D34 Whether there is a regular inspection, maintenance, maintenance system	0.0206
			D35 Whether equipped with professional facility maintenance personnel	0.0113
			D36 Whether there is a regular training and education system	0.0109
			D37Whether there is a communication and feedback mechanism	0.0089
			D38 Whether social sports instructors are available	0.0112
		C7 emergency security (0.0744)	D39 Whether to develop emergency measures and rescue plans	0.0288
			D40 Whether it is equipped with emergency rescue equipment, and regular maintenance	0.0199
			D41 Whether community residents are insured	0.0105
			D42 Whether the equipment purchased is insured	0.0147
	B4environmental factor (0.0988)	C8 internal environment (0.0682)	D43 Whether the lighting is up to standard	0.0127
			D44 The content of easily accessible materials on the surface is qualified, such as lead, cadmium, etc	0.0177
			D45 Indoor pollutant content is qualified, such as ammonia, formaldehyde, benzene, etc	0.0209
			D46 Whether the state of hygiene is good	0.0133
		C9 external environment (0.0275)	D47 Whether climate and weather can cause equipment failure	0.0046
			D48 Whether the air quality index is qualified	0.0133
			D49 Whether there are other sources of pollution, such as chemical plants and garbage cans	0.0087

### Discussion and analysis of safety evaluation index system

Equipment factor:Among the first-level indicators, the equipment factor carries the highest weight, signifying that equipment safety significantly influences the safety of public sports facilities in urban communities. Equipment safety primarily relies on three factors: facility site, facility structure, and security facilities. The five indicators of C1 facility site address site safety, a crucial factor for resident safety. This includes ground conditions, layout planning, safety buffer zone, surface layer material selection, per capita physical exercise area, and equipment—all clearly specified in national standards. The ``General Safety Requirements for Outdoor Fitness Equipment'' (GB 19,272–2011) outlines evaluation standards for D1, D3, and D4. The reasonableness of layout planning for D2 facilities can be assessed using the ``Sports Building Design Code'' (JGJ31–2003). As per the national standard, the per capita sports area for D5 is 2.6 square meters. Equipment quantity is evaluated based on the Basic Configuration Standards of National Public Sports Facilities.

C2 facility structure evaluates the structure, function, and appearance of equipment components. The overall integrity of the D6 instrument structure impacts equipment safety; D7 instrument surface safety concerns sharp corners and edges threatening resident safety; D8 equipment's key parts include main components like bearings; D9 equipment's vulnerable parts, checked daily by management personnel, are those daily contact points for residents; Aging of D10 facilities may cause instrument loosening; D11 assesses if human body contact gaps meet safety standards, ensuring fingers (gap < 8 cm) and head (gap < 30 cm) safety; D12-D13 markings on equipment indicate quality compliance; D6, D7, D8, D9, D10, D12, D13, D14 evaluates through visual inspection, while D11 and D15 require instrument measurement, following ``General Safety Requirements for Outdoor Fitness Equipment'' (GB 19,272–2011) standards.

The setup of C3 security facilities is rational and reliable, aiding in accident prevention and reduction. It encompasses four key aspects: setup layout, facility structure reliability, placement of safety signs, and the rest area. D15–16 can be assessed using the ``Code for Design of Sports Buildings'' (JGJ31–2019) standard, D17 based on ``Safety Signs and Guidelines for their Use'' (GB 2849–2008), and D18 as per the ``Configuration and Management of Outdoor Fitness Facilities for Public Sports Facilities'' (GB/T 34,290–2017) standard.

Human factor:The primary objective of the evaluation system is to guarantee the personal and property safety of individuals. Personnel factors are categorized into staff and users. Staff possessing pertinent sports knowledge, a heightened safety awareness, and regular engagement in safety training can effectively guide residents in using fitness equipment, diminishing the likelihood of sports-related injuries. Assessing whether staff members have emergency response capabilities to promptly and effectively address emergencies is crucial in minimizing accidents. The safety of equipment users encompasses various aspects, with the foremost being the individual's physical health. Conditions like heart disease, joint issues, or ongoing illnesses that are incompatible with exercise increase the risk of accidental injuries. Factors such as a weak sense of self-protection, non-compliance with safety rules, unreasonable exercise loads, and inappropriate attire can lead to falls and sprains during exercise. These indicators are evaluated through inquiries and visual observations, while exercise loads can be measured using heart rate monitoring devices like heart rate watches and chest straps. This evaluation aligns with the ``Safe Use of Public Sports Facilities'' standard (GB/T 37,913–2019).

Management factor:Management factors play a pivotal role in the overall safety and user satisfaction of public sports facilities in urban communities. Effective management can mitigate accidents and potential risks. These factors can be categorized into two aspects: system and emergency support. The system should be established and enhanced, encompassing equipment maintenance, inspection systems, and regular safety training for staff. Additionally, considerations include the presence of specialized equipment maintenance personnel and social sports instructors. Evaluation indicators are assessed through inquiries and measurements, aligning with the ``Public Sports Facilities Outdoor Fitness Facilities Configuration and Management'' standard (GB/T 34,290–2017). Emergency protection focuses on formulating emergency plans, providing emergency rescue equipment, and ensuring residents and equipment insurance. These aspects aim to safeguard the rights and interests of residents, primarily assessed through inquiries and measurements.

Environmental factor:Environmental factors are crucial indicators that cannot be overlooked in ensuring the safety of community public sports facilities. Although their weight ratio is small in the overall system, an inadequate environment can still pose risks to our physical health. These factors are categorized into C8 internal environment and C9 external environment. The internal environment encompasses factors and conditions within the facility, including lighting, pollutant content, and health conditions. The external environment refers to conditions and factors surrounding the facility, such as air quality and weather conditions. The key to managing the internal and external environments lies in considering the location and level of control over these factors. The internal environment is typically directly controlled and managed, while the external environment is not directly overseen by facility managers.

The internal environment's lighting facilities, including illuminance and glare index, are crucial for safety. Substandard lighting can reduce visibility, jeopardize safety, and insufficient illuminance may lead to unclear vision, elevating the risk of accidents and visual health issues. Surface materials easily accessible on the facility, exceeding standards, can penetrate the skin, causing severe issues such as headaches, diarrhea, and liver damage. If indoor pollutant content is below standards, it may result in chronic health problems for residents, including respiratory diseases, allergies, and even cancer. Poor hygiene fosters bacteria growth, impacting health. C8 internal environmental indicators require measurement using specialized instruments. Evaluation criteria for these indicators can be found in ``Stadium Lighting Design and Testing Standards'' (JGJ153–2016), ``Outdoor Fitness Equipment Safety Requirements'' (GB 19,272–2011), and ``Indoor Air Bacteria Total Health Standard'' (GB/T 17,093–1997). External environmental indicators involve inspecting for other pollution sources and assessing climate impacts on the facility, which can be done visually. The air quality index requires instrument measurement, following the ``Indoor Air Quality Standard'' (GB/T 18,883–2002).

## Method validation

### Establish a safety evaluation model

This study utilizes the safety evaluation indices of urban community public sports facilities at various hierarchical levels as evaluation factors. The overall objective is designated as A, with the primary-level indicators represented by factor Bi, the secondary-level indicators by factor Ci, and the tertiary-level indicators by factor Di. The model incorporates a three-tiered set of factors that must be constructed separately, with the comprehensive evaluation factor sets for each level presented in Table 5.

**Table 5:** Fuzzy evaluation factor set.

Set of Factors/layer	Set of Factors			
First layer	*A*={B1,B2,B3,B4}			
Second layer	B1={C1,C2,C3}	B2={C4,C5}	B3={C6,C7}	B4={C8,C9}
Third layer	C1={D1,D2,D3}	C2={D4,D5,D6,D7,D8,D9,D10,D11,D12,}		C3={D13,D14,D15,D16}
	C4={D17,D18,D19,D20}	C5={D21,D22,D23,D24,D25,D26,D27,D28}		
	C6={D29,D30,D31,D32,D33,D34,D35}	C7={D36,D37,D38,D39}		
	C8={D40,D41,D42,D43}	C9={D44,D45,D46}		

Based on the calculated indicator weights from Section 4, a weight set, denoted as W, is established, comprising three hierarchical layers of weight sets.

The first layer:

$W_{A}=\left\{Bi\right\}=\left\{0.4724,0.2527,0.1761,0.0988\right\}$

The second layer:

$Wb_{1}=\left\{0.3954,0.4196,0.1566\right\}$; $Wb_{2}=\left\{0.2758,0.6917\right\}$; $Wb_{3}=\left\{0.5622,0.4225\right\}$; $Wb_{4}=\left\{0.6902,0.2783\right\}$

The third layer:

$W_{C1}=\left\{0.5509,\;0.3533,\;0.0905\right\}$; $Wc_{2}=\left\{0.2316,\;0.0767,\;0.1726,\;0.0903,\;0.1271,\;0.0863,\;0.0671,\;0.0444,\;0.2180\right\}$; $Wc_{3}=\left\{0.3689,\;0.2703,\;0.2622,\;0.1473\right\}$; $Wc_{4}$={0.3802,0.2439,0.1822,0.2296}; $Wc_{5}=\left\{0.2872,\;0.0938,\;0.1693,\;0.1367,\;0.1373,\;0.0938,0.0584,\;0.0692\right\}$; $Wc_{6}=\;\left\{0.1778,\;0.1960,\;0.2081,\;0.1141,\;0.1101,\;0.0899,\;0.1131\right\}$; $Wc_{7}=\left\{0.3871,\;0.2675,\;0.1411,\;0.1976\right\}$; $Wc_{8}=\left\{0.1862,\;0.2595,\;0.3065,\;0.1950\right\}$; $Wc_{9}=\left\{0.1673,\;0.4836,\;0.3164\right\}$

Using the safety evaluation indices at various levels for urban community public sports facilities as evaluation factors, an evaluation grade set V is constructed, defined as V={V1, V2, V3, V4, V5}, where V1 represents ``Very Safe,'' V2 denotes ``Relatively Safe,'' V3 indicates ``Moderate,'' V4 stands for ``Hazardous,'' and V5 signifies ``Highly Hazardous.''

The evaluation membership matrix is constructed, with the membership set defined as follows:

$$Ri={\left\{r_{i1},r_{i2},r_{i3},\ldots ,r_{ij}\right\}}_{,}$$

Where the indicator Ri represents the degree of membership of the i th evaluation factor to each evaluation grade Vj within the assessment set. The $r_{ij}$ is:

$$r_{ij}=\frac{P_{ij}}{P}$$

Where the Pij is The number of individuals selecting the Vj grade for the i th indicator, P is The total number of participants in the evaluation

The evaluation membership matrix formed by the assessment sets of the m factors is given by:

$$R=\left[\begin{array}{ccc} r_{11} & \cdots & r_{1n}\\ \vdots & \ddots & \vdots \\ r_{m1} & \cdots & r_{mn} \end{array}\right]$$

By combining the membership matrix and the weight set, the synthesized fuzzy comprehensive evaluation result vector Z is calculated as follows:

$$Z=W*R=\left(W_{1},\;W_{2},\;\cdots W_{m}\right)*\left[\begin{array}{ccc} r_{11} & \cdots & r_{1n}\\ \vdots & \ddots & \vdots \\ r_{m1} & \cdots & r_{mn} \end{array}\right]$$

Safety Evaluation of Urban Community Public Sports Facilities

Design of the assessment methodology:This method employs random sampling to select two communities in City W, one from the central urban area and the other from a non-central district, as evaluation samples. Community A is located in the central urban area, while Community B is situated in a non-central district. The survey participants are primarily community residents.

The evaluation data were compiled based on residents' responses to the ``Survey on the Feasibility of the Safety Evaluation Index System for Public Sports Facilities in Urban Communities of City W.'' Details can be found in Table 6. Both offline and online questionnaires were used, with 100 questionnaires distributed in Community A, 98 of which were returned, and 4 deemed invalid. In Community B, 110 questionnaires were distributed, with 104 returned and 5 considered invalid.

**Table 6:** Survey locations and sample sizes.

Community Name	Total Sample Size	Total Number of Valid Samples	Questionnaire Validity Rate
A	100	94	94 %
B	110	99	90 %
Total	210	193	91.9 %

**Fig. 5:**
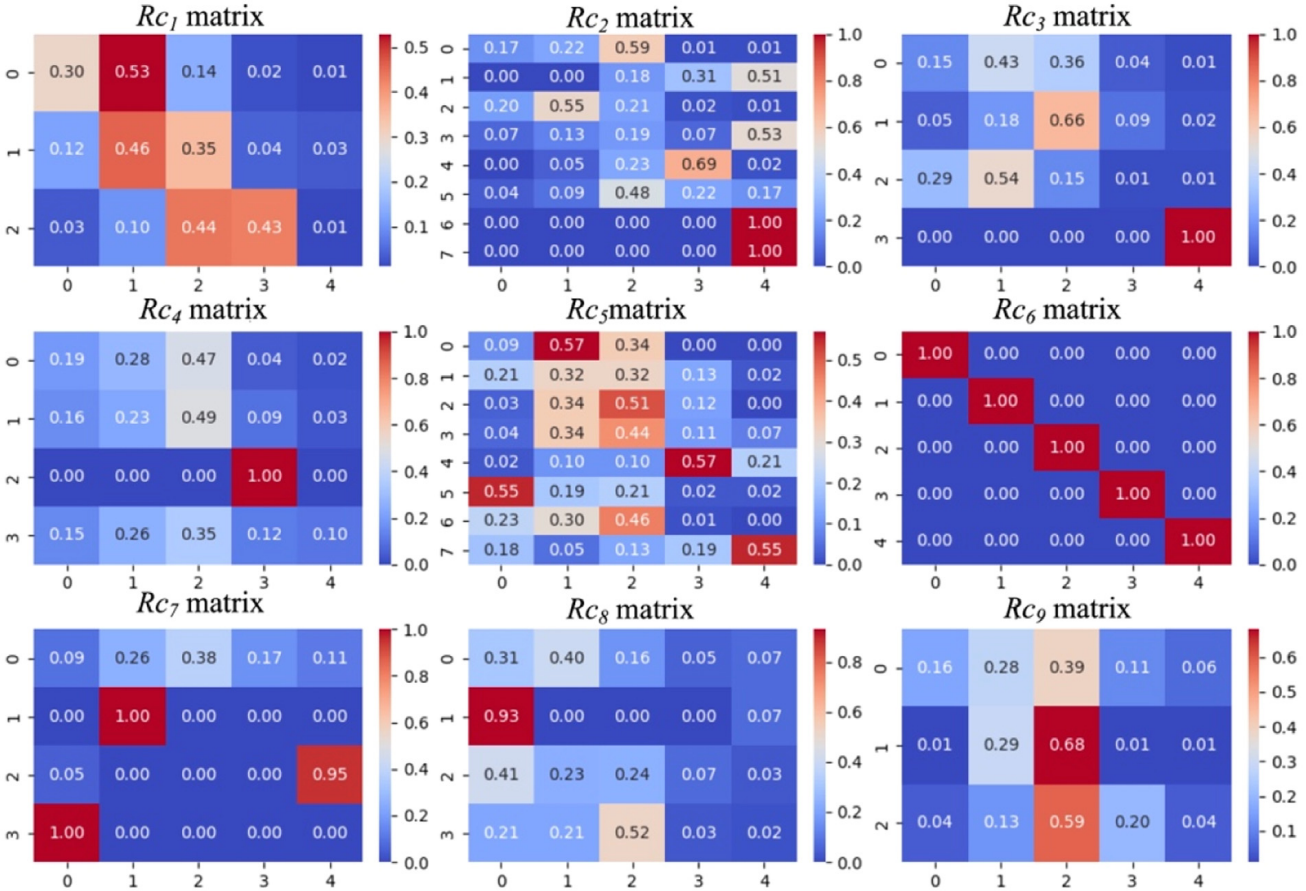
The matrix of $Rc_{1}$ to $Rc_{9}.$

A fuzzy evaluation matrix was established, using Community A as an example. The collected questionnaires were statistically analyzed, and the evaluation data were further processed for analysis. Fig. 5

The membership degrees for the tertiary indicators were calculated based on the statistical values from the evaluation opinion table of Community A. The fuzzy relationship matrices between the tertiary indicators $C_{1}$ to $C_{9}$ and the evaluation set V, denoted as $Rc_{1}$ to $Rc_{9}$, are shown as follows:

### Fuzzy comprehensive evaluation

Firstly, the fuzzy comprehensive judgement of the second-level indicators is calculated from the fuzzy matrix and weight table, and then the fuzzy matrix of each first-level indicator is derived according to the above calculation results. According to the steps of fuzzy comprehensive judgement of the second-level indicators, the fuzzy comprehensive judgement of the first-level indicators is carried out. Finally, the fuzzy comprehensive judgement of the target level indicators is carried out according to the steps of the fuzzy comprehensive judgement of the first level indicators. The safety of public sports facilities in Community B is calculated as shown in Table 6.

The fuzzy comprehensive evaluation results for the primary indicators of Community A are as follows: the equipment factor is categorized as ``Relatively Safe,'' the personnel factor is classified as ``Moderate,'' the institutional factor is rated as ``Relatively Safe,'' and the environmental factor is deemed ``Very Safe.'' Thus, the overall safety level of public sports facilities in Community A is assessed to be ``Relatively Safe.''

In contrast, the fuzzy comprehensive evaluation results for the primary indicators of Community B are as follows: the equipment factor is classified as ``Moderate,'' the personnel factor is rated as ``Moderate,'' the institutional factor is deemed ``Hazardous,'' and the environmental factor is assessed as ``Moderate.'' Consequently, the overall safety level of public sports facilities in Community B is determined to be ‘Moderate’. Table 8

The overall scores for the secondary indicators of Communities A and B are presented in Table 7.

**Table 7:** Safety assessment of public sports facilities in communities A and B.

Community	Index	Very safe	safer	normal	Danger	Very dangerous	Risk level
A	Target layer	0.1574	0.3487	0.2668	0.1382	0.0925	safer
	Primary index	Equipment factors	0.1538	0.3557	0.2954	0.0935	safer
		Personnel factors	0.1345	0.3036	0.3419	0.1659	normal
		institutional factors	0.1012	0.4827	0.0621	0.2680	safer
		Environmental factors	0.3338	0.1918	0.3030	0.0493	Very safe
B	Target layer	0.0846	0.1626	0.3528	0.3081	0.0905	normal
	Primary index	Equipment factors	0.0606	0.1248	0.4033	0.2925	normal
		Personnel factors	0.0670	0.2586	0.4207	0.2000	normal
		institutional factors	0.0926	0.1212	0.0932	0.6213	Danger
		Environmental factors	0.2299	0.1719	0.3999	0.1009	normal

**Table 8:** Evaluation table of secondary indicators for communities A and B.

Community	Index	Very safe	safer	normal	Danger	Very dangerous	Risk level
A	C1 Facility Site	0.2104	0.4635	0.2406	0.0641	0.0170	safer
	C2 Facility Structure	0.1142	0.2807	0.3465	0.1469	0.2219	normal
	C3 Security facilities	0.1449	0.3489	0.3505	0.0417	0.1590	normal
	C4 staff	0.1457	0.2222	0.3786	0.2469	0.0379	normal
	C5 User	0.1363	0.3503	0.3434	0.1414	0.0802	safer
	C6 System	0	0.5819	0	0.4272	0	safer
	C7 Emergency support	0.2395	0.3681	0.1471	0.0658	0.1766	safer
	C8 Internal environment	0.4657	0.1859	0.2048	0.0366	0.0443	Very safe
	C9 External Environment	0.0443	0.2282	0.5808	0.0865	0.0275	normal
B	C1 Facility Site	0.0537	0.1440	0.3579	0.3498	0.0794	normal
	C2 Facility Structure	0.0621	0.1212	0.4786	0.2691	0.1730	normal
	C3 Security facilities	0.0851	0.1083	0.3934	0.2638	0.1891	normal
	C4 staff	0.1007	0.2115	0.3979	0.2643	0.0409	normal
	C5 User	0.0610	0.2896	0.4495	0.1838	0.0567	normal
	C6 System	0	0.1778	0	0.8313	0	Danger
	C7 Emergency support	0.2191	0.0503	0.2206	0.3643	0.1337	Danger
	C8 Internal environment	0.3174	0.1896	0.3518	0.0775	0.0541	normal
	C9 External Environment	0.0389	0.1475	0.5644	0.1702	0.0367	normal

The public sports facilities in Community A are rated as ``Moderate'' in terms of structural integrity, security measures, staffing, and external environment. In contrast, the facility venues, user demographics, institutional framework, and emergency support systems are assessed as ``Relatively Safe,'' while the internal environment is deemed ``Very Safe.''

In Community B, the public sports facilities receive a ``Moderate'' rating across the categories of facility venues, structural integrity, security measures, staffing, user demographics, internal environment, and external environment. However, the institutional framework and emergency support systems are classified as ``Hazardous.''

### Comprehensive analysis of the 4M theory results

Analysis of Equipment Factors in the ``4M'' Theory: in Community A, the equipment factor is rated as ``Relatively Safe,'' primarily due to the facility venues being in a ``Relatively Safe'' condition. This can be attributed to the community's location in the central urban area, where sports facilities are relatively superior. Urban centers typically receive more investment and resources for infrastructure development.

In contrast, Community B's facility venues are rated as ``Moderate,'' likely due to its location in a non-central district, where inadequate management and maintenance, along with insufficient fitness equipment, may contribute to this lower rating. However, both Communities A and B have ``Moderate'' ratings for structural integrity and security measures of the facilities. A thorough analysis of the underlying causes for these ratings is essential.

(1) Risks Associated with Layout Planning

A well-designed layout can effectively mitigate safety issues in urban community public sports facilities at their source. Proper planning enhances the controllability of the venue and ensures a safer environment for users, providing residents with a secure space for physical activity. During the questionnaire survey, it was observed that Community B's public sports facilities exhibit poor layout planning. Specifically, the facilities are positioned near the entrance and exit of the neighborhood and are located too close to the roadway, posing significant safety risks.

(2) Risks Related to Facility Quality

The quality of facilities is directly linked to the safety of residents. Both Communities A and B exhibit several common issues regarding the quality of their equipment. For instance, the investigation revealed that none of the equipment in either community bears the ``NSCC'' certification mark, which significantly undermines the quality assurance of the sports equipment.

Moreover, prolonged use has led to structural deficiencies in the equipment, compromising both its functionality and safety. Issues such as wear and tear, aging, and non-compliance with stability standards have also been observed, collectively increasing the risk of accidental injuries for users.

(3) Inadequate Security Facilities

The proper and reliable installation of security facilities plays a critical role in preventing and mitigating accidents. These facilities primarily include layout design, structural reliability, placement of safety signs, and the provision of rest areas.

The investigation revealed significant deficiencies in security facilities in both communities. In Community A, the security facilities are old and partially damaged, while Community B lacks such facilities entirely. Regarding the placement of safety signs, Community A demonstrates relatively reasonable arrangements, whereas Community B's placement is problematic, with instances of obstruction that may exacerbate safety risks.

Additionally, neither community provides areas for users to rest, change clothes, or access drinking water. This lack of amenities could lead to incidents such as heatstroke during hot summer months, further compromising user safety.

Analysis of Human Factors in the ``4M'' Theory: Additionally, neither community provides areas for users to rest, change clothes, or access drinking water. This lack of amenities could lead to incidents such as heatstroke during hot summer months, further compromising user safety.

Unsafe human behavior is the direct cause of safety incidents involving community public sports facilities. These unsafe behaviors can be categorized into two groups: those of staff members and those of facility users.

For staff members, their knowledge of sports and physical activities, practical skills, safety awareness, and participation in regular safety training are critical factors. These attributes determine their ability to provide professional guidance to facility users, thereby reducing the risk of exercise-related injuries. Additionally, staff emergency response skills play a vital role in minimizing the consequences of safety incidents involving community residents.

For facility users, factors such as physical condition, attire, exercise intensity, and self-protection awareness significantly influence their safety.

Both Communities A and B received a ``Moderate'' rating for human factors. This evaluation can be attributed to the following reasons:

1) Staff Training and Awareness: Staff members in both communities do not regularly participate in safety training, and their overall safety awareness is relatively low.
2) Demographics of Facility Users: The majority of users in these communities are elderly individuals and children, who generally have limited self-rescue abilities and weak self-protection awareness in the event of accidents.
3) Health Risks Among the Elderly: Elderly users often face health-related risks during physical activities due to their poorer physical condition.
4) Lack of Preparation and Rule Adherence: Most residents do not perform proper warm-up exercises before using the facilities, nor do they follow safety guidelines or rules, increasing their risk of injury.

Addressing these issues requires targeted strategies, including enhancing staff training programs, promoting safety awareness among users, and implementing community-wide safety education initiatives.

Analysis of Management Factors in the ``4M'' Theory: Effective management is crucial for mitigating potential risks, ensuring the long-term sustainability of sports facilities, and safeguarding user safety. The risks associated with the management of urban community public sports facilities primarily stem from deficiencies in institutional systems and emergency response mechanisms.

From the perspective of institutional systems, inadequate or ineffective maintenance can lead to equipment damage or hazardous conditions. Therefore, it is essential to establish robust maintenance strategies, including regular inspections, repairs, and equipment replacement. Key aspects include the presence of safety inspection systems, equipment management protocols, and regular maintenance and servicing schedules. Additionally, providing regular safety education and training for staff can enhance their safety awareness. Employing professional maintenance personnel and certified social sports instructors can further reduce potential risks.

The investigation revealed that, compared to Community B, Community A has a slightly more comprehensive institutional framework. However, Community B received a ``Hazardous'' rating for institutional factors due to the absence of essential safety inspection systems, equipment management protocols, and regular maintenance schedules.

Moreover, neither community has professional maintenance personnel or certified social sports instructors. Both lack well-established systems for regular staff training and education. The absence of a comprehensive institutional framework results in aging and worn equipment not being repaired or replaced promptly. Over time, this exacerbates the deterioration of the facilities, threatens their safety, and perpetuates a vicious cycle of poor management in the sector.

Addressing these issues requires the implementation of standardized management protocols, allocation of professional resources, and continuous training programs to ensure the safety and sustainability of community public sports facilities.

Analysis of Environmental Factors in the ``4M'' Theory: Effective management is crucial for mitigating potential risks, ensuring the long-term sustainability of sports facilities, and safeguarding user safety. The risks associated with the management of urban community public sports facilities primarily stem from deficiencies in institutional systems and emergency response mechanisms.

Environmental factors encompass the natural and human-induced conditions that directly or indirectly influence a system, organization, process, or scenario. These factors are critical for effective planning, management, and decision-making as they can significantly impact system performance, sustainability, and safety.

Extreme weather conditions, such as high temperatures, cold climates, and humidity, can damage facilities and lead to equipment failures. Poor air quality, noise pollution, proximity to waste disposal areas, and inadequate lighting can negatively affect users' health and safety. In indoor fitness spaces, excessive levels of ammonia, formaldehyde, benzene, or heavy metals like lead and cadmium on facility surfaces pose additional health hazards.

Hygiene and cleanliness are also essential considerations. Accumulated garbage around facilities can foster the spread of bacteria or viruses, compromising public health, particularly when shared equipment is used. Proper maintenance of sanitation standards helps prevent disease transmission and supports safer use of facilities.

### Community analysis

Community A: The internal environment of Community A is relatively safe, with adequate lighting, safe levels of contact materials, and acceptable indoor air pollutant levels. However, the overall sanitation is rated as ``Moderate,'' and the facilities are more vulnerable to weather conditions, air quality fluctuations, and other pollution sources.

Community B: Community B faces more significant challenges. Its lighting conditions are poor, and the sanitation status is concerning, further heightening risks for facility users.

Considering these risks, a comprehensive approach to environmental management, including improved sanitation practices, proper material selection, robust facility maintenance, and mitigation of environmental pollutants, is essential to ensure the health and safety of community residents.

## Limitations

None.

## Ethics statements

This study involved human subjects through expert interviews and community surveys. Informed consent was obtained from all participants prior to their involvement in the study. Participants were provided with detailed information regarding the purpose, methodology, and confidentiality of the study. They were assured that their participation was voluntary and that they could withdraw at any time without consequences. All data collected were anonymized and used solely for research purposes.

## CRediT authorship contribution statement

**Xingyue Wu:** Methodology, Data curation. **Yuan Lu:** Supervision. **Chuyuan Ma:** Investigation, Funding acquisition, Formal analysis, Data curation, Conceptualization.

## Declaration of competing interest

The authors declare that they have no known competing financial interests or personal relationships that could have appeared to influence the work reported in this paper.

## Data Availability

Data will be made available on request.

## Appendix Supplementary materials

Image, application 1
