# Supplementary material for: An evaluation method for safety applied to public sports facilities in urban communities
Source: MethodsX. 2025 Mar 8;14:103256. doi: 10.1016/j.mex.2025.103256 (PMC11957586; doi:10.1016/j.mex.2025.103256)
Supplement: Supplementary file 1 [file mmc1.docx]

**Research on construction of safety evaluation index system of public sports facilities in urban communities**

**Expert Consultation Questionnaire (First round)**

Dear expert,

Hello! I am a student at the School of School of Safety Science and Emergency Management, Wuhan University of Technology. Now I am completing the paper "Research on the Construction of Safety Evaluation index System of Public sports Facilities in Urban Communities". In order to scientifically and accurately evaluate the safety of public sports facilities in communities, this study has specially designed this questionnaire. Based on your academic background, you, as an expert in this survey, are invited to provide valuable opinions on the rationality of the specific indicators established in this paper based on the factors affecting the safety of public sports facilities in communities. Your opinions will serve as an important basis for me to establish an indicator system. I really hope to get your help, and I would like to express my heartfelt thanks for your help!

Please check whether this indicator can be used as the corresponding indicator. If yes, please select from the indicator options or type "√". If you have any modification suggestions, please fill in your valuable suggestions in the questionnaire.

# *1.Theoretical basis for index construction:*

1.1 National policies, sports facilities safety standards, safety accidents collation and analysis

1.2 Safety system theory is a comprehensive methodology used to analyze, evaluate and manage the safety of systems. The main research objects are accident system and safety system. The accident system involves four elements, namely: unsafe behavior of Men, unsafe state of Machine, poor Medium factors, and inadequate management measures, which are usually referred to as "4M" elements (as shown in Figure 2 below). The establishment of the safety evaluation system of public sports facilities in urban communities needs a unified basic framework with great guiding significance. Based on the basic framework of "equipment-man-management-environment", indicators are unified through literature research, on-site inspection and review, historical accident analysis and expert consultation to form an organic whole

According to the characteristics of public sports facilities in urban communities, this paper combines the safety of public sports facilities in urban communities with the new "management-man-machine-environment" system engineering theory, and builds a scientific, systematic, operable and effective safety evaluation index system of public sports facilities in urban communities. In the system engineering theory, "human" refers to the personnel who use the facilities; "Machine" means the equipment of the community sports facility itself; "Environment" refers to the special conditions in which "man" and "machine" coexist, mainly including the facility environment and the surrounding environment; "Management" refers to the community management level.

# *2. The overall indicator framework (easy for experts to preview, no need to fill in)*

| [goal level](javascript:;) | Primary index | Second level  index | Three-level index |
| --- | --- | --- | --- |
| Safety of public sports facilities in urban communities | equipment  factor | facility  site | 1) Whether the ground condition of the site is safe, such as smooth, non-slip, etc.; 2) Whether the facilities are placed reasonably; 3) Whether the safety buffer zone of the site meets the general requirements of the project rules; 4) Whether the selection of surface layer materials meets the standard; 5) Whether the per capita physical exercise area and equipment meet the standard; |
|  |  | facility  structure | 1) Whether the overall structure of the instrument is intact; 2) Whether the surface of the instrument is safe, such as fading, edges, sharp corners, etc. 3) Whether the key parts of the equipment are intact; 4) Whether the vulnerable parts of the equipment are in good condition; 5) Whether the facilities are aged; 6) Whether the gap between the easy contact areas of the human body is qualified; 7) Whether there is an "NSCC" certification mark; 8) Whether the equipment is configured with information instructions, such as use instructions, service life, etc.; |
|  |  | security  facility | 1) Whether the security facilities are set reasonably; 2) Whether the structure of security facilities is reliable; 3) Whether the safety signs and signage are set correctly; 4) Whether to set a safe area for users to rest, change clothes, etc.; |
|  | human factor | staff | 1) Whether it has relevant professional knowledge and practical ability; 2) Whether you have a high degree of safety awareness; 3) Whether to participate in safety training regularly; 4) Whether it has the emergency handling ability; |
|  |  | user | 1) Whether the state of health is good; 2) Whether they have safety awareness; 3) Compliance with rules and safety guidelines; 4) Whether the exercise load is reasonable; 5) Whether they have the ability to respond to emergencies; 6) Whether there is a sense of supervision and mutual assistance; |
|  | management factors | institutional system | 1) Whether there is a safety inspection system; 2) Whether there is a equipment management system; 3) Whether the system is regularly inspected, maintained, maintained and updated; 4) Whether to implement the performance evaluation system; 5) Whether to establish a health management system; 6) Whether it is equipped with professional facility maintenance personnel; 7) Whether there is a regular training and education system; 8) Whether there is a communication and feedback mechanism; 9) Whether it is equipped with social sports instructors; 10) Whether professional security personnel are equipped |
|  |  | emergency security | 1) Whether to develop emergency measures and rescue plans; 2) Whether it is equipped with emergency rescue equipment, and regular maintenance; 3) Whether community citizens are insured; 4) Whether the community manager has taken out relevant liability insurance according to law; 5) Whether the purchased equipment is insured; |
|  | environmental factor | internal environment | 1) Whether the lighting facilities meet the standards; 2) The content of easily accessible materials on the surface is qualified, such as lead, cadmium, etc.; 3) Whether the indoor pollutant content is qualified, such as ammonia, formaldehyde, benzene, etc.; |
|  |  | [external environment](javascript:;) | 1) Whether climate and weather will cause equipment failure; 2) Whether the air quality index is qualified; 3) Whether there are other sources of pollution around, such as chemical plants, garbage cans, etc.; |

# *3. Essential information*

| Name |  | Work unit |  |
| --- | --- | --- | --- |
| Professional qualifications |  | Job |  |
| Education background |  | Basic work years |  |

# *4. Do you agree with the following first-level indicators for the safety evaluation of public sports facilities in urban communities?*

| Primary index | strongly agree | Compare  agree | ordinary | Compare  disagree | very  disagree |
| --- | --- | --- | --- | --- | --- |
| equipment factor |  |  |  |  |  |
| human factor |  |  |  |  |  |
| management factors |  |  |  |  |  |
| environmental factor |  |  |  |  |  |
| Your opinion on whether the content contained in each level indicator should be added or removed |  | | | | |

# *5. Do you agree with the following secondary indicators of safety evaluation of public sports facilities in urban communities?*

| Primary index | Second level  index | strongly agree | Compare  agree | ordinary | Compare  disagree | very  disagree |
| --- | --- | --- | --- | --- | --- | --- |
| equipment factor | facility  site |  |  |  |  |  |
|  | facility  structure |  |  |  |  |  |
|  | security  facility |  |  |  |  |  |
| human factor | staff |  |  |  |  |  |
|  | user |  |  |  |  |  |
| management factors | institutional system |  |  |  |  |  |
|  | emergency security |  |  |  |  |  |
| environmental factor | internal environment |  |  |  |  |  |
|  | external environment |  |  |  |  |  |
| Your comments on whether the content contained in each secondary indicator should be added or removed |  | | | | | |

# *6. Do you agree with the following three indexes of safety evaluation of public sports facilities in urban communities?*

| Second level  index | Three-level index | strongly agree | Compare  agree | ordinary | Compare  disagree | very  disagree | suggestions on revision |
| --- | --- | --- | --- | --- | --- | --- | --- |
| facility  site | 1)Whether the ground condition of the site is safe, such as smooth, non-slip, etc. |  |  |  |  |  |  |
|  | 2)Whether the facilities are placed reasonably |  |  |  |  |  |  |
|  | 3）Whether the safety buffer zone of the site meets the general requirements of the project rules |  |  |  |  |  |  |
|  | 4)Whether the selection of surface layer materials meets the standard |  |  |  |  |  |  |
|  | 5)Whether the per capita physical exercise area and equipment meet the standard; |  |  |  |  |  |  |
| facility  structure | 1) Whether the overall structure of the instrument is intact |  |  |  |  |  |  |
|  | 2）Whether the surface of the instrument is safe, such as fading, edges, sharp corners, etc. |  |  |  |  |  |  |
|  | 3）Whether the key parts of the equipment are intact |  |  |  |  |  |  |
|  | 4）Whether the vulnerable parts of the equipment are in good condition |  |  |  |  |  |  |
|  | 5）Whether the facilities are aged |  |  |  |  |  |  |
|  | 6）Whether the gap between the easy contact areas of the human body is qualified |  |  |  |  |  |  |
|  | 7)Whether there is an "NSCC" certification mark |  |  |  |  |  |  |
|  | 8)Whether the equipment is configured with information instructions, such as use instructions, service life, etc. |  |  |  |  |  |  |
| security  facility | 1) Whether the security facilities are set reasonably |  |  |  |  |  |  |
|  | 2) Whether the structure of security facilities is reliable |  |  |  |  |  |  |
|  | 3) Whether the safety signs and signage are set correctly |  |  |  |  |  |  |
|  | 4) Whether to set a safe area for users to rest, change clothes, etc. |  |  |  |  |  |  |
| staff | 1）Whether it has relevant professional knowledge and practical ability |  |  |  |  |  |  |
|  | 2)Whether you have a high degree of safety awareness |  |  |  |  |  |  |
|  | 3）Whether to participate in safety training regularly |  |  |  |  |  |  |
|  | 4）Whether it has the emergency handling ability |  |  |  |  |  |  |
| user | 1）Whether the state of health is good |  |  |  |  |  |  |
|  | 2）Whether they have safety awareness |  |  |  |  |  |  |
|  | 3）Compliance with rules and safety guidelines |  |  |  |  |  |  |
|  | 4）Whether the exercise load is reasonable |  |  |  |  |  |  |
|  | 5）Whether they have the ability to respond to emergencies |  |  |  |  |  |  |
|  | 6）Whether there is a sense of supervision and mutual assistance |  |  |  |  |  |  |
| institutional system | 1)Whether there is a safety inspection system |  |  |  |  |  |  |
|  | 2)Whether there is a equipment management system |  |  |  |  |  |  |
|  | 3) Whether the system is regularly inspected, maintained, maintained and updated |  |  |  |  |  |  |
|  | 4) Whether to implement the performance evaluation system |  |  |  |  |  |  |
|  | 5) Whether to establish a health management system |  |  |  |  |  |  |
|  | 6) Whether it is equipped with professional facility maintenance personnel |  |  |  |  |  |  |
|  | 7)Whether there is a regular training and education system |  |  |  |  |  |  |
|  | 8)Whether there is a communication and feedback mechanism |  |  |  |  |  |  |
|  | 9）Whether it is equipped with social sports instructors |  |  |  |  |  |  |
|  | 10)Whether professional security personnel are equipped |  |  |  |  |  |  |
| emergency security | 1) Whether to develop emergency measures and rescue plans; |  |  |  |  |  |  |
|  | 2) Whether it is equipped with emergency rescue equipment, and regular maintenance |  |  |  |  |  |  |
|  | 3)Whether community citizens are insured |  |  |  |  |  |  |
|  | 4) Whether the community manager has taken out relevant liability insurance according to law |  |  |  |  |  |  |
|  | 5）Whether the purchased equipment is insured |  |  |  |  |  |  |
| internal environment | 1)Whether the lighting facilities meet the standards |  |  |  |  |  |  |
|  | 2)The content of easily accessible materials on the surface is qualified, such as lead, cadmium, etc. |  |  |  |  |  |  |
|  | 3）Whether the indoor pollutant content is qualified, such as ammonia, formaldehyde, benzene, etc. |  |  |  |  |  |  |
| external environment | 1)Whether climate and weather will cause equipment failure |  |  |  |  |  |  |
|  | 2）Whether the air quality index is qualified |  |  |  |  |  |  |
|  | 3)Whether there are other sources of pollution around, such as chemical plants, garbage cans, etc.; |  |  |  |  |  |  |
| Your opinion on whether the content contained in each level indicator should be added or removed |  | | | | | | |

**Expert judgment basis questionnaire**

| Your basis for judging the above indicators and the extent of their impact  Each criterion can only select one influence level and mark it "√". | | | | | |
| --- | --- | --- | --- | --- | --- |
| Influence  degree  Judgment basis | big | | middle | | small |
| Practical experience |  | |  | |  |
| Theoretical analysis |  | |  | |  |
| Understanding of domestic and foreign counterparts |  | |  | |  |
| Intuitive feeling |  | |  | |  |
| Your familiarity with the content of the survey | | | | | |
| familiarity | Very familiar | More familiar | General familiarity | Not familiar | Very unfamiliar |
| Expert  self-assessment |  |  |  |  |  |

**Research on construction of safety evaluation index system of public sports facilities in urban communities**

**Expert Consultation Questionnaire (Second round)**

Dear expert,

Hello! I am a 2021 graduate student at the School of School of Safety Science and Emergency Management, Wuhan University of Technology. Now I am completing the paper "Research on the Construction of Safety Evaluation index System of Public sports Facilities in Urban Communities". In order to scientifically and accurately evaluate the safety of public sports facilities in communities, this study has specially designed this questionnaire. Based on your academic background, you, as an expert in this survey, are invited to provide valuable opinions on the rationality of the specific indicators established in this paper based on the factors affecting the safety of public sports facilities in communities. Your opinions will serve as an important basis for me to establish an indicator system. I really hope to get your help, and I would like to express my heartfelt thanks for your help!

Please check whether this indicator can be used as the corresponding indicator. If yes, please select from the indicator options or type "√". If you have any modification suggestions, please fill in your valuable suggestions in the questionnaire.

# *1. Theoretical basis for index construction:*

1.1 National policies, sports facilities safety standards, safety accidents collation and analysis

1.2 Safety system theory is a comprehensive methodology used to analyze, evaluate and manage the safety of systems. The main research objects are accident system and safety system. The accident system involves four elements, namely: unsafe behavior of Men, unsafe state of Machine, poor Medium factors, and inadequate management measures, which are usually referred to as "4M" elements (as shown in Figure 2 below). The establishment of the safety evaluation system of public sports facilities in urban communities needs a unified basic framework with great guiding significance. Based on the basic framework of "equipment-man-management-environment", indicators are unified through literature research, on-site inspection and review, historical accident analysis and expert consultation to form an organic whole

According to the characteristics of public sports facilities in urban communities, this paper combines the safety of public sports facilities in urban communities with the new "management-man-machine-environment" system engineering theory, and builds a scientific, systematic, operable and effective safety evaluation index system of public sports facilities in urban communities. In the system engineering theory, "human" refers to the personnel who use the facilities; "Machine" means the equipment of the community sports facility itself; "Environment" refers to the special conditions in which "man" and "machine" coexist, mainly including the facility environment and the surrounding environment; "Management" refers to the community management level.

# *2. The overall indicator framework (easy for experts to preview, no need to fill in)*

| [goal level](javascript:;) | Primary index | Second level  index | Three-level index |
| --- | --- | --- | --- |
| Safety of public sports facilities in urban communities | equipment  factor | facility  site | 1) Whether the ground condition of the site is safe, such as smooth, non-slip, etc.; 2）Whether the facility layout planning is reasonable; 3) Whether the safety buffer zone of the site meets the general requirements of the project rules; 4) Whether the selection of surface layer materials meets the standard; 5) Whether the per capita physical exercise area and equipment meet the standard; |
|  |  | facility  structure | 1) Whether the overall structure of the instrument is intact; 2) Whether the surface of the instrument is safe, such as fading, edges, sharp corners, etc. 3) Whether the key parts of the equipment are intact; 4) Whether the vulnerable parts of the equipment are in good condition; 5) Whether the facilities are aged; 6) Whether the gap between the easy contact areas of the human body is qualified; 7) Whether there is an "NSCC" certification mark; 8) Whether the equipment is configured with information instructions, such as use instructions, service life, etc.; |
|  |  | security  facility | 1) Whether the security facilities are set reasonably; 2) Whether the structure of security facilities is reliable; 3) Whether the safety signs and signage are set correctly; 4) Whether to set a safe area for users to rest, change clothes, etc.; |
|  | human factor | staff | 1) Whether it has relevant professional knowledge and practical ability; 2) Whether you have a high degree of safety awareness; 3) Whether to participate in safety training regularly; 4) Whether it has the emergency handling ability; |
|  |  | user | 1) Whether the state of health is good; 2) Whether you have a sense of self-protection; 3) Compliance with rules and safety guidelines; 4) Whether the exercise load is reasonable; 5) Whether they have the ability to respond to emergencies; 6) Whether there is a sense of supervision and mutual assistance; |
|  | management factors | institutional system | 1) Whether there is a safety inspection system; 2) Whether there is a equipment management system; 3) Whether the system is regularly inspected, maintained, maintained and updated; 4) Whether it is equipped with professional facility maintenance personnel; 5) Whether there is a regular training and education system; 6) Whether there is a communication and feedback mechanism; 7) Whether it is equipped with social sports instructors |
|  |  | emergency security | 1) Whether to develop emergency measures and rescue plans; 2) Whether it is equipped with emergency rescue equipment, and regular maintenance; 3) Whether community citizens are insured; 4) Whether the community manager has taken out relevant liability insurance according to law; 5) Whether the purchased equipment is insured; |
|  | environmental factor | internal environment | 1) Whether the lighting facilities meet the standards; 2) The content of easily accessible materials on the surface is qualified, such as lead, cadmium, etc.; 3) Whether the indoor pollutant content is qualified, such as ammonia, formaldehyde, benzene, etc.; 4) whether the health condition is good; |
|  |  | [external environment](javascript:;) | 1) Whether climate and weather will cause equipment failure; 2) Whether the air quality index is qualified; 3) Whether there are other sources of pollution around, such as chemical plants, garbage cans, etc.; |

# *3. Essential information*

| Name |  | Work unit |  |
| --- | --- | --- | --- |
| Professional qualifications |  | Job |  |
| Education background |  | Basic work years |  |

# *4. Do you agree with the following first-level indicators for the safety evaluation of public sports facilities in urban communities?*

| Primary index | strongly agree | Compare  agree | ordinary | Compare  disagree | very  disagree |
| --- | --- | --- | --- | --- | --- |
| equipment factor |  |  |  |  |  |
| human factor |  |  |  |  |  |
| management factors |  |  |  |  |  |
| environmental factor |  |  |  |  |  |
| Your opinion on whether the content contained in each level indicator should be added or removed |  | | | | |

# *5. Do you agree with the following secondary indicators of safety evaluation of public sports facilities in urban communities?*

| Primary index | Second level  index | strongly agree | Compare  agree | ordinary | Compare  disagree | very  disagree |
| --- | --- | --- | --- | --- | --- | --- |
| equipment factor | facility  site |  |  |  |  |  |
|  | facility  structure |  |  |  |  |  |
|  | security  facility |  |  |  |  |  |
| human factor | staff |  |  |  |  |  |
|  | user |  |  |  |  |  |
| management factors | institutional system |  |  |  |  |  |
|  | emergency security |  |  |  |  |  |
| environmental factor | internal environment |  |  |  |  |  |
|  | external environment |  |  |  |  |  |
| Your comments on whether the content contained in each secondary indicator should be added or removed |  | | | | | |

# *6.Do you agree with the following three indexes of safety evaluation of public sports facilities in urban communities?*

| Second level  index | Three-level index | strongly agree | Compare  agree | ordinary | Compare  disagree | very  disagree | suggestions on revision |
| --- | --- | --- | --- | --- | --- | --- | --- |
| facility  site | 1)Whether the ground condition of the site is safe, such as smooth, non-slip, etc. |  |  |  |  |  |  |
|  | 2)Whether the facility layout planning is reasonable |  |  |  |  |  |  |
|  | 3）Whether the safety buffer zone of the site meets the general requirements of the project rules |  |  |  |  |  |  |
|  | 4)Whether the selection of surface layer materials meets the standard |  |  |  |  |  |  |
|  | 5)Whether the per capita physical exercise area and equipment meet the standard; |  |  |  |  |  |  |
| facility  structure | 1) Whether the overall structure of the instrument is intact |  |  |  |  |  |  |
|  | 2）Whether the surface of the instrument is safe, such as fading, edges, sharp corners, etc. |  |  |  |  |  |  |
|  | 3）Whether the key parts of the equipment are intact |  |  |  |  |  |  |
|  | 4）Whether the vulnerable parts of the equipment are in good condition |  |  |  |  |  |  |
|  | 5）Whether the facilities are aged |  |  |  |  |  |  |
|  | 6）Whether the gap between the easy contact areas of the human body is qualified |  |  |  |  |  |  |
|  | 7)Whether there is an "NSCC" certification mark |  |  |  |  |  |  |
|  | 8)Whether the equipment is configured with information instructions, such as use instructions, service life, etc. |  |  |  |  |  |  |
| security  facility | 1) Whether the security facilities are set reasonably |  |  |  |  |  |  |
|  | 2) Whether the structure of security facilities is reliable |  |  |  |  |  |  |
|  | 3) Whether the safety signs and signage are set correctly |  |  |  |  |  |  |
|  | 4) Whether to set a safe area for users to rest, change clothes, etc. |  |  |  |  |  |  |
| staff | 1）Whether it has relevant professional knowledge and practical ability |  |  |  |  |  |  |
|  | 2)Whether you have a high degree of safety awareness |  |  |  |  |  |  |
|  | 3）Whether to participate in safety training regularly |  |  |  |  |  |  |
|  | 4）Whether it has the emergency handling ability |  |  |  |  |  |  |
| user | 1）Whether the state of health is good |  |  |  |  |  |  |
|  | 2）Whether you have a sense of self-protection |  |  |  |  |  |  |
|  | 3）Compliance with rules and safety guidelines |  |  |  |  |  |  |
|  | 4）Whether the exercise load is reasonable |  |  |  |  |  |  |
|  | 5）Whether they have the ability to respond to emergencies |  |  |  |  |  |  |
|  | 6）Whether there is a sense of supervision and mutual assistance |  |  |  |  |  |  |
| institutional system | 1)Whether there is a safety inspection system |  |  |  |  |  |  |
|  | 2)Whether there is a equipment management system |  |  |  |  |  |  |
|  | 3) Whether the system is regularly inspected, maintained, maintained and updated |  |  |  |  |  |  |
|  | 4) Whether it is equipped with professional facility maintenance personnel |  |  |  |  |  |  |
|  | 5)Whether there is a regular training and education system |  |  |  |  |  |  |
|  | 6)Whether there is a communication and feedback mechanism |  |  |  |  |  |  |
|  | 7）Whether it is equipped with social sports instructors |  |  |  |  |  |  |
| emergency security | 1) Whether to develop emergency measures and rescue plans; |  |  |  |  |  |  |
|  | 2) Whether it is equipped with emergency rescue equipment, and regular maintenance |  |  |  |  |  |  |
|  | 3)Whether community citizens are insured |  |  |  |  |  |  |
|  | 4) Whether the community manager has taken out relevant liability insurance according to law |  |  |  |  |  |  |
|  | 5）Whether the purchased equipment is insured |  |  |  |  |  |  |
| internal environment | 1)Whether the lighting facilities meet the standards |  |  |  |  |  |  |
|  | 2)The content of easily accessible materials on the surface is qualified, such as lead, cadmium, etc. |  |  |  |  |  |  |
|  | 3）Whether the indoor pollutant content is qualified, such as ammonia, formaldehyde, benzene, etc. |  |  |  |  |  |  |
|  | 4）whether the health condition is good |  |  |  |  |  |  |
| external environment | 1)Whether climate and weather will cause equipment failure |  |  |  |  |  |  |
|  | 2）Whether the air quality index is qualified |  |  |  |  |  |  |
|  | 3)Whether there are other sources of pollution around, such as chemical plants, garbage cans, etc.; |  |  |  |  |  |  |
| Your opinion on whether the content contained in each level indicator should be added or removed |  | | | | | | |

**Expert judgment basis questionnaire**

| Your basis for judging the above indicators and the extent of their impact  Each criterion can only select one influence level and mark it "√". | | | | | |
| --- | --- | --- | --- | --- | --- |
| Influence  degree  Judgment basis | big | | middle | | small |
| Practical experience |  | |  | |  |
| Theoretical analysis |  | |  | |  |
| Understanding of domestic and foreign counterparts |  | |  | |  |
| Intuitive feeling |  | |  | |  |
| Your familiarity with the content of the survey | | | | | |
| familiarity | Very familiar | More familiar | General familiarity | Not familiar | Very unfamiliar |
| Expert  self-assessment |  |  |  |  |  |

**Research on construction of safety evaluation index system of public sports facilities in urban communities**

**Expert Consultation Questionnaire (Third round)**

Dear expert,

Hello! I am a 2021 graduate student at the School of School of Safety Science and Emergency Management, Wuhan University of Technology. Now I am completing the paper "Research on the Construction of Safety Evaluation index System of Public sports Facilities in Urban Communities". In order to scientifically and accurately evaluate the safety of public sports facilities in communities, this study has specially designed this questionnaire. Based on your academic background, you, as an expert in this survey, are invited to provide valuable opinions on the rationality of the specific indicators established in this paper based on the factors affecting the safety of public sports facilities in communities. Your opinions will serve as an important basis for me to establish an indicator system. I really hope to get your help, and I would like to express my heartfelt thanks for your help!

Please check whether this indicator can be used as the corresponding indicator. If yes, please select from the indicator options or type "√". If you have any modification suggestions, please fill in your valuable suggestions in the questionnaire.

# *1. Theoretical basis for index construction:*

1.1 National policies, sports facilities safety standards, safety accidents collation and analysis

1.2 Safety system theory is a comprehensive methodology used to analyze, evaluate and manage the safety of systems. The main research objects are accident system and safety system. The accident system involves four elements, namely: unsafe behavior of Men, unsafe state of Machine, poor Medium factors, and inadequate management measures, which are usually referred to as "4M" elements (as shown in Figure 2 below). The establishment of the safety evaluation system of public sports facilities in urban communities needs a unified basic framework with great guiding significance. Based on the basic framework of "equipment-man-management-environment", indicators are unified through literature research, on-site inspection and review, historical accident analysis and expert consultation to form an organic whole

According to the characteristics of public sports facilities in urban communities, this paper combines the safety of public sports facilities in urban communities with the new "management-man-machine-environment" system engineering theory, and builds a scientific, systematic, operable and effective safety evaluation index system of public sports facilities in urban communities. In the system engineering theory, "human" refers to the personnel who use the facilities; "Machine" means the equipment of the community sports facility itself; "Environment" refers to the special conditions in which "man" and "machine" coexist, mainly including the facility environment and the surrounding environment; "Management" refers to the community management level.

# *2. The overall indicator framework (easy for experts to preview, no need to fill in)*

| [goal level](javascript:;) | Primary index | Second level  index | Three-level index |
| --- | --- | --- | --- |
| Safety of public sports facilities in urban communities | equipment  factor | facility  site | 1) Whether the ground condition of the site is safe, such as smooth, non-slip, etc.; 2）Whether the facility layout planning is reasonable; 3) Whether the safety buffer zone of the site meets the general requirements of the project rules; 4) Whether the selection of surface layer materials meets the standard; 5) Whether the per capita physical exercise area and equipment meet the standard; |
|  |  | facility  structure | 1) Whether the overall structure of the instrument is intact; 2) Whether the surface of the instrument is safe, such as fading, edges, sharp corners, etc. 3) Whether the key parts of the equipment are intact; 4) Whether the vulnerable parts of the equipment are in good condition; 5) Whether the facilities are aged; 6) Whether the gap between the easy contact areas of the human body is qualified; 7) Whether there is an "NSCC" certification mark; 8) Whether the equipment is configured with information instructions, such as use instructions, service life, etc.;9) Whether the equipment meets the stability requirements; 10) Whether the static load of the main components meets the standard |
|  |  | security  facility | 1) Whether the security facilities are set reasonably; 2) Whether the structure of security facilities is reliable; 3) Whether the safety signs and signage are set correctly; 4) Whether to set a safe area for users to rest, change clothes, etc.; |
|  | human factor | staff | 1) Whether it has relevant professional knowledge and practical ability; 2) Whether you have a high degree of safety awareness; 3) Whether to participate in safety training regularly; 4) Whether it has the emergency handling ability; |
|  |  | user | 1) Whether the state of health is good; 2) Whether you have a sense of self-protection; 3) Compliance with rules and safety guidelines; 4) Whether the exercise load is reasonable; 5) Whether they have the ability to respond to emergencies; 6) Whether there is a sense of supervision and mutual assistance; 7) Whether the dress is suitable for sports; 8) Whether to warm up before exercise |
|  | management factors | institutional system | 1) Whether there is a safety inspection system; 2) Whether there is a equipment management system; 3) Whether the system is regularly inspected, maintained, maintained and updated; 4) Whether it is equipped with professional facility maintenance personnel; 5) Whether there is a regular training and education system; 6) Whether there is a communication and feedback mechanism; 7) Whether it is equipped with social sports instructors |
|  |  | emergency security | 1) Whether to develop emergency measures and rescue plans; 2) Whether it is equipped with emergency rescue equipment, and regular maintenance; 3) Whether community citizens are insured; 4) Whether the community manager has taken out relevant liability insurance according to law; 5) Whether the purchased equipment is insured; |
|  | environmental factor | internal environment | 1) Whether the lighting facilities meet the standards; 2) The content of easily accessible materials on the surface is qualified, such as lead, cadmium, etc.; 3) Whether the indoor pollutant content is qualified, such as ammonia, formaldehyde, benzene, etc.; 4) whether the health condition is good; |
|  |  | [external environment](javascript:;) | 1) Whether climate and weather will cause equipment failure; 2) Whether the air quality index is qualified; 3) Whether there are other sources of pollution around, such as chemical plants, garbage cans, etc.; |

# *3. Essential information*

| Name |  | Work unit |  |
| --- | --- | --- | --- |
| Professional qualifications |  | Job |  |
| Education background |  | Basic work years |  |

# *4. Do you agree with the following first-level indicators for the safety evaluation of public sports facilities in urban communities?*

| Primary index | strongly agree | Compare  agree | ordinary | Compare  disagree | very  disagree |
| --- | --- | --- | --- | --- | --- |
| equipment factor |  |  |  |  |  |
| human factor |  |  |  |  |  |
| management factors |  |  |  |  |  |
| environmental factor |  |  |  |  |  |
| Your opinion on whether the content contained in each level indicator should be added or removed |  | | | | |

# *5. Do you agree with the following secondary indicators of safety evaluation of public sports facilities in urban communities?*

| Primary index | Second level  index | strongly agree | Compare  agree | ordinary | Compare  disagree | very  disagree |
| --- | --- | --- | --- | --- | --- | --- |
| equipment factor | facility  site |  |  |  |  |  |
|  | facility  structure |  |  |  |  |  |
|  | security  facility |  |  |  |  |  |
| human factor | staff |  |  |  |  |  |
|  | user |  |  |  |  |  |
| management factors | institutional system |  |  |  |  |  |
|  | emergency security |  |  |  |  |  |
| environmental factor | internal environment |  |  |  |  |  |
|  | external environment |  |  |  |  |  |
| Your comments on whether the content contained in each secondary indicator should be added or removed |  | | | | | |

# *6. Do you agree with the following three indexes of safety evaluation of public sports facilities in urban communities?*

| Second level  index | Three-level index | strongly agree | Compare  agree | ordinary | Compare  disagree | very  disagree | suggestions on revision |
| --- | --- | --- | --- | --- | --- | --- | --- |
| facility  site | 1)Whether the ground condition of the site is safe, such as smooth, non-slip, etc. |  |  |  |  |  |  |
|  | 2)Whether the facility layout planning is reasonable |  |  |  |  |  |  |
|  | 3）Whether the safety buffer zone of the site meets the general requirements of the project rules |  |  |  |  |  |  |
|  | 4)Whether the selection of surface layer materials meets the standard |  |  |  |  |  |  |
|  | 5)Whether the per capita physical exercise area and equipment meet the standard; |  |  |  |  |  |  |
| facility  structure | 1) Whether the overall structure of the instrument is intact |  |  |  |  |  |  |
|  | 2）Whether the surface of the instrument is safe, such as fading, edges, sharp corners, etc. |  |  |  |  |  |  |
|  | 3）Whether the key parts of the equipment are intact |  |  |  |  |  |  |
|  | 4）Whether the vulnerable parts of the equipment are in good condition |  |  |  |  |  |  |
|  | 5）Whether the facilities are aged |  |  |  |  |  |  |
|  | 6）Whether the gap between the easy contact areas of the human body is qualified |  |  |  |  |  |  |
|  | 7)Whether there is an "NSCC" certification mark |  |  |  |  |  |  |
|  | 8)Whether the equipment is configured with information instructions, such as use instructions, service life, etc. |  |  |  |  |  |  |
|  | 9)Whether the equipment meets the stability requirements; |  |  |  |  |  |  |
|  | 10) Whether the static load of the main components meets the standard |  |  |  |  |  |  |
| security  facility | 1) Whether the security facilities are set reasonably |  |  |  |  |  |  |
|  | 2) Whether the structure of security facilities is reliable |  |  |  |  |  |  |
|  | 3) Whether the safety signs and signage are set correctly |  |  |  |  |  |  |
|  | 4) Whether to set a safe area for users to rest, change clothes, etc. |  |  |  |  |  |  |
| staff | 1）Whether it has relevant professional knowledge and practical ability |  |  |  |  |  |  |
|  | 2)Whether you have a high degree of safety awareness |  |  |  |  |  |  |
|  | 3）Whether to participate in safety training regularly |  |  |  |  |  |  |
|  | 4）Whether it has the emergency handling ability |  |  |  |  |  |  |
| user | 1）Whether the state of health is good |  |  |  |  |  |  |
|  | 2）Whether you have a sense of self-protection |  |  |  |  |  |  |
|  | 3）Compliance with rules and safety guidelines |  |  |  |  |  |  |
|  | 4）Whether the exercise load is reasonable |  |  |  |  |  |  |
|  | 5）Whether they have the ability to respond to emergencies |  |  |  |  |  |  |
|  | 6）Whether there is a sense of supervision and mutual assistance |  |  |  |  |  |  |
|  | 7) Whether the dress is suitable for sports |  |  |  |  |  |  |
|  | 8) Whether to warm up before exercise |  |  |  |  |  |  |
| institutional system | 1)Whether there is a safety inspection system |  |  |  |  |  |  |
|  | 2)Whether there is a equipment management system |  |  |  |  |  |  |
|  | 3) Whether the system is regularly inspected, maintained, maintained and updated |  |  |  |  |  |  |
|  | 4) Whether it is equipped with professional facility maintenance personnel |  |  |  |  |  |  |
|  | 5)Whether there is a regular training and education system |  |  |  |  |  |  |
|  | 6)Whether there is a communication and feedback mechanism |  |  |  |  |  |  |
|  | 7）Whether it is equipped with social sports instructors |  |  |  |  |  |  |
| emergency security | 1) Whether to develop emergency measures and rescue plans; |  |  |  |  |  |  |
|  | 2) Whether it is equipped with emergency rescue equipment, and regular maintenance |  |  |  |  |  |  |
|  | 3)Whether community citizens are insured |  |  |  |  |  |  |
|  | 4) Whether the community manager has taken out relevant liability insurance according to law |  |  |  |  |  |  |
|  | 5）Whether the purchased equipment is insured |  |  |  |  |  |  |
| internal environment | 1)Whether the lighting facilities meet the standards |  |  |  |  |  |  |
|  | 2)The content of easily accessible materials on the surface is qualified, such as lead, cadmium, etc. |  |  |  |  |  |  |
|  | 3）Whether the indoor pollutant content is qualified, such as ammonia, formaldehyde, benzene, etc. |  |  |  |  |  |  |
|  | 4）whether the health condition is good |  |  |  |  |  |  |
| external environment | 1)Whether climate and weather will cause equipment failure |  |  |  |  |  |  |
|  | 2）Whether the air quality index is qualified |  |  |  |  |  |  |
|  | 3)Whether there are other sources of pollution around, such as chemical plants, garbage cans, etc.; |  |  |  |  |  |  |
| Your opinion on whether the content contained in each level indicator should be added or removed |  | | | | | | |
